# Risk of disability pension in first and second generation immigrants: the role of age and region of birth in a prospective population-based study from Sweden

**DOI:** 10.1186/s12889-017-4944-x

**Published:** 2017-12-04

**Authors:** D. Di Thiene, M. Helgesson, K. Alexanderson, G. La Torre, J. Tiihonen, E. Mittendorfer-Rutz

**Affiliations:** 1 Division of Insurance Medicine, Department of Clinical Neuroscience, Karolinska Institutet, SE-171 77 Stockholm, Sweden; 2 Department of Public Health and Infectious Diseases, Sapienza University of Rome, Rome, Italy; 3 Center for Psychiatric Research, Department of Clinical Neuroscience, Karolinska Institutet, SE-171 77 Stockholm, Sweden

**Keywords:** Migration, Disability pension, Sick leave

## Abstract

**Background:**

In several countries, immigrants have higher disability pension (DP) rates than natives. Reasons for this are poorly understood. The aim of this study was to investigate if the risk of diagnosis-specific DP differed in first, second, and second/intermediate generation immigrants compared to natives, in general and across regions of birth, and stratified by age.

**Methods:**

A population-based prospective cohort study of all 3,507,055 individuals aged 19–50 years and living in Sweden in 2004 with a 6-year follow-up period. Hazard ratios (HR) and 95% confidence intervals (CI) for mental and somatic DP were estimated by Cox regression for first, second, and second/intermediate generation immigrants compared to natives, across regions of birth and stratified by age.

**Results:**

After multivariate adjustment, HRs for both mental and somatic DP were higher at follow-up in the first generation compared to natives: mental HR 1.17 (CI 1.12–1.22) and somatic 1.15 (1.09–1.22) for individuals <35 years; 1.74 (1.69–1.79) and 1.70 (1.66–1.74) ≥35 years (median), respectively. Immigrants born in Europe outside EU25, and countries outside Europe had particularly elevated HRs. Also in the second generation, HRs were higher in mental 1.29 (1.21–1.37) and somatic DP: 1.30 (1.19–1.42) in those <35 years; and 1.18 (1.10–1.27); and 1.10 (1.03–1.17) for those ≥35 years, respectively. Among second generation immigrants there were no strong differences in HRs between regions of birth.

**Conclusions:**

Compared to natives, the risk of DP was higher in first and second generation immigrants. Higher estimates were seen for immigrants from Europe outside EU25 and from the rest of the world in the first generation. No considerable differences in estimates regarding mental or somatic DP diagnoses were found.

## Background

Sweden is now a multicultural society; more than 25% of the residents are first or second generation migrants, a rate that is expected to increase in the following years [1]. According to a United Nation report, most of the immigrants arriving to Sweden are of working age (median age of 41 years), which emphasises the importance of their possible labour market participation [2]. Nowadays, early exit from the labour market due to work disability, i.e., disability pension (DP), constitutes a considerable public health problem in a number of OECD countries. Both in Sweden and Norway, DP is reported to be more common among immigrants than in the native population [3–5].

Previous studies report that socioeconomic status plays an important role in the risk of DP [3, 6]. Higher rates of low socioeconomic status among first generation immigrants have been shown to contribute to the differences in risk of DP between first generation immigrants and natives [4, 5, 7]. Additionally, higher levels of morbidity can be present in immigrants compared to natives and thus involve higher DP risk, as showed in several studies [4, 7–10]. Based on these reported findings, more knowledge is needed considering both socio-economic status and morbidity factors when studying associations of immigrant status and future DP.

Among the many potential risk factors for DP, age is one of the most important. Previous studies showed that the risk of DP is higher in higher ages [3, 11, 12]. Focusing on immigrants, a Norwegian study found a higher risk of DP among immigrants in comparison to natives, but when adjusting for age at granted DP, the differences disappeared [5]. To the best of our knowledge, no study to date has investigated if age has differential associations with subsequent DP risk across generations of immigrants.

Another important factor of DP is the diagnosis: today mental diagnoses and somatic diagnoses (mostly musculoskeletal diagnoses, are the two major DP diagnostic groups in OECD countries [3, 13–16]. Previous studies have shown that immigrants have a higher risk for depressive disorders but no previous studies have investigated if there are differences in immigrants’ risk of being granted DP due to mental diagnoses and specific somatic diagnoses, compared to native Swedes.

In order to evaluate labour market participation among the offspring of immigrants, reflecting the integration in the new country, this study includes not only the first generations but also second/intermediate (one parent born abroad) and second generation immigrants (two parents born abroad). Previous studies have shown that the risk of morbidity, such as schizophrenia, suicide, or acute myocardial infarction differs on the basis of the generation of immigrants [17–20]. Still, to date knowledge on the associations between first generation and particularly second generation immigrants and subsequent DP is limited [21, 22].

Region of birth represents another aspect involved in the heterogeneity of immigrant groups. Studies from Sweden showed different rates of DP for immigrants on the basis of region of birth particularly in first generation immigrants from Northern European countries and immigrants from outside Europe (i.e., Middle East and North Africa) [4, 5, 7]. The higher risk of DP in immigrants from these regions has been shown to be to some extent explained by differences in age and working conditions [5, 23]. Previous studies, however, did not investigate the association between region of birth of immigrants with regard to subsequent DP.

In summary, the current study will fill important knowledge gaps related to the association between different migrant subgroups and subsequent DP by taking both age, the heterogeneity of the migrant subgroups, and several DP diagnoses into account. In specific, this study scrutinizes both country of birth as well as first and second generation of migrants for DP due to mental as well as somatic diagnoses.

The aim of this study was to investigate if the risk of DP due to mental or somatic diagnoses differed in first, second, and second/intermediate generation immigrants compared to natives, in general, across regions of birth, and stratified by age.

## Methods

### Design

This is a population-based prospective cohort study with a 6-year follow-up, based on data linked from several nationwide Swedish registers. The study base included all individuals between 19 and 50 years of age and resident in Sweden at 31 December 2004 (*N* = 3,751,056).

### Register data

Nationwide register data, linked at individual level (based on the unique personal number), from the following authorities were used: 1) Statistics Sweden: used for identifying the cohort, for information on socio-demographic factors: age, sex, education, family situation, type of residential area, country of birth, and unemployment (a measure of labor market marginalization). The multi-generation register was used for information on country of birth of the included individuals’ parents; 2) the Social Insurance Agency: for information on DP (date and diagnoses); and 3) the National Board of Health and Welfare: for dates on inpatient and specialized outpatient care due to mental and somatic disorders as well as dates of mortality.

### Study population

In order to reach the best coverage of parental information on the country of birth (valid for the second generation immigrants) from the Multi-generation register of Statistics Sweden, the upper age limit was set at 50 years [24].

Moreover, individuals with missing information on own country of birth (*N* = 251), maternal country of birth (*N* = 5073), or paternal country of birth (*N* = 41,138) were excluded. Individuals already on DP during 2004 (*N* = 179,822) were also excluded. Moreover, individuals born abroad with parents born in Sweden (*N* = 17,717), for example, international adoptees, i.e., a group with specific characteristics, were not included [16]. The final study population consisted of 3,507,055 individuals at risk of subsequent DP. Of these, 946,632 (26.9%) individuals were first, second/intermediate, or second generation immigrants.

### Disability pension in Sweden

In Sweden, all residents between 19 and 64 years with a long-term or permanent reduction of work capacity due to disease or injury can be granted DP by the Social Insurance Agency. A process is necessary before being granted DP: repeated medical and work-capacity assessments from physicians are performed, along with rehabilitation measures. For individuals aged 19–29 years, temporary DP is possible if the work capacity is reduced for at least 1 year or if upper-secondary education is not completed due to disease or injury. DP can be granted for 25, 50, 75 or 100% of ordinary working hours [25].

### Immigration status and categorization of region of birth

First-generation immigrant status was defined as being born outside Sweden, with both parents born outside Sweden. Individuals were categorized in the group of second generation immigrants, when born in Sweden with both parents born outside Sweden. An additional category was the “second/intermediate” generation, defined as born in Sweden and having one parent born in Sweden and the other parent born abroad [18, 26, 27]. Individuals born in Sweden, with both parents born in Sweden (here called natives) were defined as the reference population [4, 28].

Moreover, regions of birth were classified into five subgroups: “Sweden”, “Nordic countries” (Finland, Denmark, Norway, and Iceland); “EU-25+” (countries included in the European Union in 2006 except Sweden and “Nordic countries” plus US, Canada, Australia, and New Zealand); “European countries outside EU25 and Former Soviet Union countries”; “rest of the world” (Asia, South America, Africa). The country of birth of the mother was used to define the parental region of birth related to the “second/intermediate” and the second generation. Individuals in the “second/intermediate” generation were categorized in the “Sweden” group when the mother was born in Sweden and the father was born abroad [18, 27].

### Measurement of outcome

The outcome was being granted DP due to mental or somatic diagnoses (full- or part-time) during the follow-up period. Diagnoses were categorized according to the International Classification of Diseases version 10 [29]. Mental diagnoses comprised ICD-10 codes F00–99 and somatic diagnoses comprised all remaining diagnoses in ICD-10.

### Measurement of covariates

Socio-demographic covariates, i.e., age, educational level, family situation (indicating if an individual is a) cohabiting/married or single as well as b) living with or without children at home), and type of residential area, were measured at the end of 2004. Age for the stratified analyses was dichotomized according to the median (35 years). Information on unemployment benefits during 2004 was categorized into yes/no. Moreover, to measure morbidity up to the year the follow-up began, four dichotomous variables were included, regarding inpatient care (2000–2004) and specialized outpatient care (2001–2004) due to mental (ICD-10 codes: F00–99) and somatic disorders (all other ICD-10 diagnoses). All variables were categorized as shown in Table 1.

**Table 1:** Descriptive statistics of socio-demographics, unemployment, and healthcare utilization of individuals born in Sweden (natives), first, second/intermediate^1^, and second generation immigrants, respectively. (*N* = 3,507,055)

Characteristics	Natives *n* (%)	First *n* (%)	Second *n* (%)	Second/Intermediate *n* (%)	*p* values*
All	2,564,423 (73.1)	550,424 (15.7)	122.844 (3.5)	269.364 (7.7)	
***Sociodemographic factors (2004)***					
*Sex*					*p* < 0.001
Men	1,330,709 (51.9)	270,907 (49.2)	63,781 (51.9)	139,615 (51.8)	
Women	1,233,714 (48.1)	279,517 (50.8)	59,063 (48.1)	129,749 (48.2)	
*Age (years)*					*p* < 0.001
19–24	446,325 (17.4)	80,365 (14.6)	30,262 (24.6)	54,070 (20.1)	
25–34	808,063 (31.5)	176,323 (32.0)	45,539 (37.1)	86,154 (32.0)	
35–44	852,091 (33.2)	196,524 (35.7)	33,954 (27.6)	88,490 (32.9)	
45–50	457,944 (17.9)	97,212 (17.7)	13,089 (10.7)	40,650 (15.1)	
*Education (years)*					*p* < 0.001
Compulsory school (≤9)	260,877 (10.2)	105,509 (19.2)	17,704 (14.4)	31,873 (11.8)	
High school [10–12]	1,374,598 (53.6)	218,173 (39.6)	68,313 (55.6)	141,943 (52.7)	
College/University (>12)	925,957 (36.1)	180,412 (31.0)	35,543 (28.9)	94,585 (35.1)	
Missing	2991 (0.1)	46,330 (8.4)	1284 (2.5)	963 (0.4)	
*Family situation*					*p* < 0.001
Married/cohab. With children^2^	1,095,026 (42.7)	240,901 (43.8)	41,336 (33.6)	101,047 (37.5)	
Married/cohab. Without children^2^	101,165 (3.9)	43,802 (8.0)	5251 (4.3)	9802 (3.6)	
Single without children	1,080,004 (42.1)	201,264 (36.6)	59,330 (41.7)	124,439 (46.2)	
Single with children	175,136 (6.8)	48,969 (8.9)	9040 (7.4)	20,481 (7.6)	
Young adults living at home 19-20ys	113,089 (4.4)	15,476 (2.8)	8292 (6.8)	13,594 (5.0)	
*Type of residential area* ^*3*^					*p* < 0.001
Big cities	883,937 (33.9)	301,815 (54.3)	67,070 (55.7)	124,008 (45.6)	
Medium-sized cities	942,297 (36.8)	167,324 (30.7)	37,901 (30.3)	88,573 (33.0)	
Small towns	738,189 (29.3)	81,285 (15.0)	17,873 (14.0)	56,783 (21.4)	
*Unemployed* ^*4*^	398,840 (15.6)	130,792 (23.8)	26,030 (21.2)	49,478 (18.4)	*p* < 0.001
***Healthcare***					
Inpatient, Somatic, 2000–04	599,402 (23.4)	134,630 (24.5)	28,888 (23.5)	62,717 (23.3)	*p* < 0.001
Inpatient, Mental, 2000–04	32,537 (1.3)	9275 (1.7)	2510 (2.0)	4784 (1.8)	*p* < 0.001
Outpatient, Somatic 2001–04	1,440,384 (41.1)	315,968 (57.4)	75,080 (61.1)	159,472 (59.2)	*p* < 0.001
Outpatient, Mental 2001–2004	60,276 (2.4)	16,259 (3.0)	3971 (3.2)	8143 (3.0)	*p* < 0.001

^1^Intermediate generation refers to individuals born in Sweden with one parent born in Sweden and one parent born abroad

^2^Children living at home

^3^Area of residence: big cities: Stockholm, Gothenburg and Malmö; medium-sized cities: cities with more than 90,000 inhabitants within 30 km distance from the center of the city; small cities/villages

^4^Individuals with unemployment benefits in 2004

*For differences among immigrant groups and natives

### Statistical analyses

Differences in the distributions of socio-demographic characteristics and the annual prevalence of healthcare among immigrants and natives were tested using Chi-square tests. Individuals were followed from 1 January 2005 until the event (mental or somatic DP), death, emigration, or end of follow-up (31 December 2010), whichever occurred first. After testing if the proportional hazards assumption was met, Cox proportional hazard regression models were applied. Analyses were adjusted for sex, age, educational level, family situation, residential area, and unemployment status. A second model was additionally adjusted for healthcare variables (diagnosis-specific in- and specialized outpatient healthcare) used as a proxy of morbidity. Crude and adjusted hazard ratios (HR) with 95% confidence intervals (CI) for different immigrant groups compared to natives (reference group) with regard to subsequent mental or somatic DP were calculated, across regions of birth and stratified by age. Given the heterogeneity of somatic diagnoses, additional analyses were carried out for the three most frequently occurring somatic DP diagnoses: diseases of the musculoskeletal system, injuries, and diseases of the nervous system, in order to evaluate different patterns in the associations with DP. SPSS version 20.0 was used in the analyses.

## Results

The distributions of socio-demographic and healthcare variables in natives and the three immigrant groups differed significantly (Table 1, *p* < 0.001).

In comparison to the native population, first-generation immigrants were less likely to be of younger age (17.4% vs. 14.6% of 19–24 years old), and more likely to have lower education (Table 1). The levels of unemployment and somatic specialized outpatient care were higher among immigrants of the first generation than in natives.

Compared to the native population, the second-generation immigrants were more likely to be of younger age and to have a lower educational level. Second generation immigrants also had higher rates of unemployment and of somatic specialized outpatient care than the native population.

### Mental disability pension

In total, 1.1%, 1.9%, 1.5%, and 1.4% of individuals of the native population, first, second, and second/intermediate generation, respectively, were granted DP due to mental diagnoses during the follow-up (data not shown). For first generation immigrants, the mental DP risk was higher in comparison to the native population with HRs of 1.17 for the younger group (<35 years) and 1.74 for the older ones (≥35 years) in the multivariate analyses. There were smaller age differences in the estimates for second and second/intermediate generation immigrants with regard to subsequent DP due to mental diagnoses (Table 2).

**Table 2:** Crude and multivariate hazard ratios (HR) and 95% confidence intervals (CI) for the risk of mental disability pension (DP) in the follow-up period (2005–2010) in relation to immigration status, stratified by age (median) and using natives as the reference group

*Age/* Immigration status	Population *N* (%)	Mental DP *n* (%)	Crude HR (95% CI)	Model 1 HR (95% CI)	Model 2 HR (95% CI)
*<35 years*					
Natives	1,242,910 (99.1)	11,478 (0.9)	1	1	1
First generation	253,868 (98.9)	2820 (1.1)	1.26 (1.20–1.31)	1.13 (1.08–1.17)	1.17 (1.12–1.22)
Second generation	74,709 (98.6)	1092 (1.4)	1.59 (1.49–1.69)	1.36 (1.28–1.45)	1.29 (1.21–1.37)
Second/ Intermediate generation	138,386 (98.7)	1838 (1.3)	1.44 (1.37–1.51)	1.33 (1.27–1.40)	1.21 (1.15–1.27)
*≥35 years*					
Natives	1,292,795 (98.7)	17,240 (1.3)	1	1	1
First generation	285,984 (97.4)	7752 (2.6)	2.08 (2.03–2.14)	1.88 (1.82–1.93)	1.74 (1.69–1.79)
Second generation	46,198 (98.2)	845 (1.8)	1.37 (1.28–1.47)	1.28 (1.20–1.36)	1.18 (1.10–1.27)
Second/ Intermediate generation	127,054 (98.4)	2086 (1.6)	1.23 (1.17–1.28)	1.18 (1.13–1.24)	1.13 (1.08–1.18)

Model 1: adjusted for sex, educational level, family situation, residential area, and unemployment status

Model 2: like Model 1 and additionally adjusted for healthcare variables (diagnosis-specific in- and specialized outpatient healthcare)

Nearly half of the first-generation immigrants were born in countries outside Europe. In the multivariate analyses, first generation immigrants from “European countries outside EU25 and Former Soviet Union” had a two-fold higher risk of subsequent mental DP compared to their native counterparts. The adjusted HR for first generation immigrants from the “rest of the world” was 1.46. The estimates decreased slightly after stepwise adjustment (Table 3).

**Table 3:** Crude and multivariate hazard ratios (HR) and 95% confidence intervals (CI) for the risk of mental disability pension (DP) across regions of birth in the follow-up period (2005–2010), stratified by immigration status, using natives as the reference group

Immigration status	Populationn (%)	Mental DP *n* (%)	Crude HR (95% CI)	Model 1 HR (95% CI)	Model 2 HR (95% CI)
First generation					
*Nordic*	87,263 (16.1)	1389 (1.6)	1.48 (1.41–1.55)	1.23 (1.16–1.29)	1.09 (1.03–1.15)
*EU 25+*	86,592 (16.0)	1123 (1.3)	1.22 (1.15–1.30)	1.27 (1.20–1.35)	1.26 (1.19–1.34)
*Other EU*	116,135 (21.6)	3621 (2.7)	2.50 (2.41–2.59)	2.12 (2.04–2.20)	2.06 (1.98–2.13)
*Rest*	249,862 (46.3)	4799 (1.9)	1.74 (1.68–1.79)	1.52 (1.47–1.57)	1.46 (1.43–1.52)
Second generation					
*Nordic*	65,933 (54.5)	1225 (1.8)	1.64 (1.55–1.74)	1.50 (1.42–1.59)	1.31 (1.23–1.38)
*EU 25+*	23,703 (19.6)	328 (1.4)	1.23 (1.10–1.37)	1.27 (1.14–1.41)	1.24 (1.14–1.38)
*Other EU*	21,651 (17.9)	282 (1.3)	1.15 (1.02–1.30)	1.15 (1.02–1.29)	1.18 (1.05–1.45)
*Rest*	9620 (8.0)	102 (1.0)	0.94 (0.77–1.14)	1.03 (0.84–1.24)	1.12 (0.92–1.36)
Second/ intermediate generation					
*Sweden*	130,213 (49.1)	1984 (1.5)	1.34 (1.27–1.42)	1.27 (1.20–1.34)	1.19 (1.13–1.25)
*Nordic*	89,657 (34.7)	1368 (1.5)	1.13 (1.03–1.24)	1.17 (1.06–1.28)	1.13 (1.03–1.24)
*EU 25+*	37,007 (13.1)	474 (1.3)	1.07 (0.80–1.44)	1.13 (0.85–1.52)	1.04 (0.78–1.40)
*Other EU*	3681 (1.3)	45 (1.2)	0.96 (0.73–1.24)	1.10 (0.84–1.44)	1.02 (0.78–1.34)
*Rest*	4882 (1.8)	53 (1.1)	1.34 (1.28–1.40)	1.34 (1.28–1.40)	1.22 (1.16–1.27)

Model 1: adjusted for age, sex, educational level, family situation, residential area, and unemployment status

Model 2: like Model 1 and additionally adjusted for healthcare variables (diagnosis-specific in- and specialized outpatient healthcare)

*Countries categorization*

*Nordic*: Finland, Denmark, Norway, and Iceland;

*EU-25+*: countries included in the European Union in 2006 except Sweden and “Nordic countries” plus United States, Canada, Australia, and New Zealand;

*Other EU*: European countries outside EU25 and Former Soviet Union countries;

*Rest*: Asia, South America, Africa

Half of the second-generation immigrants had parents born in Nordic countries. The multivariate adjusted HR for subsequent mental DP in this group was 1.31. For the second/intermediate-generation immigrants, having a mother from Sweden, another Nordic country, or “rest of the world” was associated with a higher risk of mental DP in comparison to natives (Table 3).

### Somatic disability pension

During the follow-up, 1.3% of the native population was granted DP due to somatic diagnoses (data not shown). These proportions were 2.1%, 1.3%, and 1.3% for the first, second and second/intermediate generation, respectively (data not shown). The HRs for subsequent somatic DP differed between the immigrant groups (HR range 0.99 to 1.70) (Table 4).

**Table 4:** Crude and multivariate hazard ratios (HR) and 95% confidence intervals (CI) for the risk of somatic disability pension (DP) in the follow-up period (2005–2010), stratified by age (median) using natives as the reference group

*Age*/ Immigration status	Populationn (%)	Somatic DP *n* (%)	Crude HR (95% CI)	Model 1 HR (95% CI)	Model 2 HR (95% CI)
*Below 35 years*					
Natives	1,247,196 (99.4)	7192 (0.6)	1	1	1
First generation	255,056 (99.4)	1632 (0.6)	1.11 (1.11–1.23)	1.11 (1.05–1.17)	1.15 (1.09–1.22)
Second generation	75,246 (99.3)	555 (0.7)	1.32 (1.18–1.41)	1.32 (1.21–1.44)	1.30 (1.19–1.42)
Second/ intermediate	139,438 (99.4)	786 (0.6)	1.01 (0.91–1.06)	1.01 (0.93–1.08)	0.99 (0.92–1.06)
*≥35 years*					
Natives	1,283,142 (97.9)	26,893 (2.1)	1	1	1
First generation	283,436 (96.5)	10,300 (3.5)	1.78 (1.74–1.82)	1.79 (1.75–1.83)	1.70 (1.66–1.74)
Second generation	45,985 (97.8)	1058 (2.2)	1.10 (1.03–1.74)	1.15 (1.08–1.22)	1.10 (1.03–1.17)
Second/ Intermediate	126,409 (97.9)	2731 (2.1)	1.03 (0.99–1.07)	1.07 (1.03–1.12)	1.04 (1.00–1.08)

Model 1: adjusted for sex, educational level, family situation, residential area, and unemployment status

Model 2: like Model 1 and additionally adjusted for healthcare variables (diagnosis-specific in- and specialized outpatient healthcare)

For younger and older first generation immigrants, the multivariate adjusted HRs related to subsequent DP due to somatic diagnoses were 1.15 and 1.70, respectively (Table 4). Also, the somatic DP risk in second generation immigrants was higher in comparison to the native population. The HR was 1.30 in the younger age group and 1.10 in the older age group. In both age groups of second/intermediate generation immigrants, there was no higher risk for somatic DP. In relation to region of birth, first generation immigrants from “European countries outside EU25 and Former Soviet Union” and from the “rest of the world” showed highest estimates (HR 2.24 and 1.54, respectively) in comparison to natives (Table 5).

**Table 5:** Crude and multivariate hazard ratios (HR) and 95% confidence intervals (CI) for the risk of somatic disability pension (DP) across regions of birth in the follow-up period (2005–2010) stratified by immigration status, using natives as the reference group

Immigration status	Population *n* (%)	Somatic DP *n* (%)	Crude HR (95% CI)	Model 1 HR (95% CI)	Model 2 HR (95% CI)
First generation					
*Nordic*	86,608 (16.1)	2044 (2.3)	1.85 (1.77–1.93)	1.31 (1.26–1.37)	1.28 (1.24–1.36)
*EU 25+*	86,549 (16.0)	1166 (1.3)	1.07 (1.01–1.14)	1.19 (1.12–1.26)	1.23 (1.16–1.30)
*Other EU*	115,637 (21.6)	3759 (3.1)	2.43 (2.35–2.52)	2.36 (2.27–2.43)	2.24 (2.17–2.33)
*Rest*	249,698 (46.3)	4963 (1.9)	1.52 (1.47–1.56)	1.68 (1.63–1.73)	1.54 (1.53–1.62)
Second generation					
*Nordic*	66,070 (54.5)	1088 (1.6)	1.23 (1.16–1.31)	1.31 (1.25–1.40)	1.24 (1.17–1.32)
*EU 25+*	23,722 (19.6)	309 (1.3)	0.98 (0.86–1.09)	1.14 (1.01–1.27)	1.15 (1.02–1.26)
*Other EU*	21,750 (17.9)	183 (0.8)	0.62 (0.54–0.74)	1.14 (0.98–1.31)	1.10 (0.95–1.27)
*Rest*	9689 (8.0)	33 (0.3)	0.25 (0.18–0.36)	0.70 (0.50–0.99)	0.76 (0.54–1.07)
Second/ intermediate generation					
*Sweden*	130,629 (49.1)	1568 (1.2)	0.89 (0.85–0.94)	1.06 (1.01–1.11)	1.04 (0.98–1.08)
*Nordic*	89,585 (34.7)	1440 (1.6)	1.18 (1.12–1.24)	1.13 (1.05–1.16)	1.10 (1.03–1.14)
*EU 25+*	37,024 (13.1)	457 (1.2)	0.91 (0.83–1.00)	0.98 (0.89–1.06)	0.97 (0.88–1.06)
*Other EU*	3699 (1.3)	27 (0.7)	0.60 (0.43–0.85)	0.84 (0.61–1.20)	0.81 (0.59–1.16)
*Rest*	4910 (1.8)	25 (0.5)	0.41 (0.29–0.57)	0.84 (0.63–1.21)	0.88 (0.67–1.28)

Model 1: adjusted for age, sex, educational level, family situation, and unemployment status

Model 2: like Model 1 and additionally adjusted for healthcare variables (diagnosis-specific in and specialized outpatient healthcare)

*Countries categorization*

*Nordic*: Finland, Denmark, Norway, and Iceland;

*EU-25+*: countries included in the European Union in 2006 except Sweden and “Nordic countries” plus US, Canada, Australia, and New Zealand;

*Other EU*: European countries outside EU25 and Former Soviet Union countries;

*Rest*: Asia, South America, Africa

In the second generation, the groups with highest risk estimates were the Nordic and the EU25+ groups (Table 5). Additional analyses were carried out for the three main somatic diagnoses: diseases of the musculoskeletal system, injuries, and diseases of the nervous system, respectively. Diseases of the musculoskeletal system and injuries showed similar patterns as the entire group of the somatic diagnoses in relation to DP among immigrants, diseases of the nervous system were the only DP diagnoses with different patterns. Here, the HRs were not significant in any the immigrant groups in comparison to natives.

## Discussion

### Main findings

In comparison to native Swedes, the future risk of DP regardless of diagnosis was higher particularly in first but also in second/intermediate and second generation immigrants. With regard to age, in the first generation immigrants, HRs were higher in the older age group in comparison to natives, and were slightly higher in the younger age group as well. Opposite age patterns were found for the second and second/intermediate generation. These associations were not explained by differences in socio-demographics, unemployment, or morbidity. When the region of birth was taken into account, the patterns of associations differed by immigrant generation. Immigrants from “European countries outside EU25 and the Former Soviet Union” and from the “rest of the world” showed highest HRs only in the first generation. On the contrary, immigrants from Nordic countries and from EU25+ showed highest risk of subsequent DP in the second generation, compared to natives.

### Discussion of findings in relation to other studies

#### Age

In first generation immigrants, the risk of DP increased with age, as showed in the general population [3, 30]. The observed age differences with regard to the first and second generation may be partly explained by the differences in length of staying in the country. As shown in a previous study, the risk of being granted DP was very low among newly arrived immigrants, but increased rapidly on a yearly basis after immigration [31]. Possible difficulties in being granted DP could be an issue in younger first-generation immigrants and, with more familiarity with the Swedish system, disappear with time. Other explanations, regarding the second generation immigrants, include the structure of the social insurance system in Sweden, i.e., that the threshold for being granted DP is somewhat lower in young adults (19–29 years of age).

#### First generation and region of birth

A higher risk of subsequent granting of DP in first generation immigrants was found than in the native population, which is in line with previous results from studies carried out in Sweden and Norway [4, 31]. We could now also show that this excess risk is regardless of DP diagnosis. When the region of birth was taken into account, first generation immigrants showed the highest rates of DP if born in “European countries outside EU25 and Former Soviet Union” and in “the rest of the world”. Immigrants from these regions were also shown to have higher rates of DP in another study [31]. This is in line with previous findings showing higher rates of morbidity and lower rates of access to healthcare and treatment in immigrants from these regions compared to natives [32–34]. These factors might be associated with cultural differences leading to marginalization and with preexisting conditions, e.g., poor health and stressful life events. This might be particularly true for immigrants from the “rest of the world” where refugees are mostly represented [35].

Pathways to DP in immigrants and natives can also differ due to divergent eligibility criteria based on the regulations of the social insurance system [36]. Of note, in Sweden DP can be granted without previous paid work. In contrary, receipt of benefits related to sickness absence, which often precedes DP, requires previous income from work or unemployment benefits [25]. As immigrants have been reported to have higher risks of labour market marginalisation, it is likely that transition to DP differs between immigrants and natives [37]. Type of work and occupation can have a role in the risk of labor market marginalization and in differences between migrants and natives. Here, first generation immigrants seem to have a higher risk to have jobs with an adverse psychosocial environment and job insecurity [38].

There might also be a possibility of discrimination both at the level of getting a job, establishing at the labor market, and at the working place. Moreover, knowledge about the social insurance system might differ between immigrants and their native counterparts. Finally, access to healthcare and adequate treatment play a crucial role to prevent the disabling process resulting in DP [39]. Differences in access to care, in the clinical manifestation and symptomatology of the underlying disease and consequently in its diagnostics, as well as differences in healthcare, have been shown for immigrants compared to the native population [40]. Moreover, adequate care in first generation immigrants might be hampered by language barriers, knowledge of the healthcare system, as well as the lack of competence in transcultural psychiatry and psychology in the healthcare settings of the host country [32].

#### Second, second/intermediate generation of immigrants and region of birth

In the second generation of immigrants the risk estimates of being granted DP were higher in comparison to natives, but lower compared to the estimates for the first generation. Different rates between first generation and second generation immigrants have been found regarding several health issues, i.e., suicide, schizophrenia, coronary heart diseases [17, 18, 41]. We could now also show these associations for DP. A possible explanation of this pattern is a lower risk to be marginalized at the labor market in the second generation compared to the first [20]. It is possible that individuals of the second generation are more assimilated in the society and better educated than their parents [42]. Previous research indicates that educational investment in Sweden, such as Swedish language education, has a positive effect on the individual chances of succeeding on the labor market [37]. Second generation immigrants do not have the same language barrier as individuals in the first generation have. Moreover, the lower risk estimates in the intermediate/s generation suggest that having a parent from Sweden seems to be a protective factors mitigating the risk of DP as previously shown in relation to other health issues [27].

Immigrants from Nordic countries showed higher risk estimates in comparison to natives in the second generation and the risk remained after adjustments. To the best of our knowledge, this has not been reported previously. Immigration from Nordic countries to Sweden is mostly from Finland [43]. Other studies showed higher rates in second generation migrants from Finland compared to natives, regarding e.g. suicide [27], mental disorders [44], or hospitalization rates due to diabetes [33]. Some of the reasons might include discrimination and adverse health behavior [44]. It has previously been noted that Finnish migrants in Sweden have fewer barriers and less economical expenses for migration. These conditions have been suggested to potentially result in the absence of the “healthy migrant effect”. Similar findings have been reported among Irish immigrants to England [45].

### Strengths and limitations

The use of a very large and population-based cohort, the prospective design and using administrative register data of high quality with practically no loss to follow up, can be mentioned among the strengths of this study [46]. This allowed for inclusion of the whole population of working ages of an entire country avoiding selection and recall bias. An additional strength is the long follow-up period and the possibility to control for several confounding factors such as sociodemographic factors, previous unemployment, and specialized healthcare. As a limitation, potential unmeasured residual confounding can be mentioned. By using information from in- and specialized outpatient care, only morbidity of greater medical severity was captured [47]. Also, the validity of DP diagnoses must be discussed, although it has not been studied. The long process of medical assessment behind granting DP might, however, assure a good validity. Moreover, it can be assumed that any misclassification has affected groups equally and thus not biased the comparisons. In 1991, Ljungdahl et al. showed a high validity of sick-leave diagnoses in comparison to diagnoses reported in medical files [48]. Another limitation is that detailed information on some covariates that might be of importance, e.g., on type of occupation and some health behaviours of importance for DP risks, was not available [49]. Finally, in order to have statistical power we categorized the immigrants into broad groups. Future studies with a focus on specific birth countries are warranted.

## Conclusions

Although the risk of disability pension due to mental or somatic diagnoses was higher among immigrants compared to natives, the risks differed between first, second, and second/intermediate generation immigrants. Moreover, patterns varied with age and region of birth and further studies are warranted to elucidate the mechanisms behind granting of disability pension in immigrant subgroups compared to natives.

## References

[1] The future population of Sweden 2016–2060. Statistics Sweden. 2016.

[2] International Migration Report 2015 (2016). United Nations, Department of Economic and Social Affairs, population division.

[3] Karlsson NE, Carstensen JM, Gjesdal S, Alexanderson KA (2008). Risk factors for disability pension in a population-based cohort of men and women on long-term sick leave in Sweden. Eur J Pub Health.

[4] Johansson B, Helgesson M, Lundberg I, Nordquist T, Leijon O, Lindberg P, et al. Work and health among immigrants and native Swedes 1990–2008: a register-based study on hospitalization for common potentially work-related disorders, disability pension and mortality. BMC Public Health. 2012;12(845). 10.1186/1471-2458-12-845 PMC3532317 23039821

[5] Claussen B, Smeby L, Bruusgaard D (2012). Disability pension rates among immigrants in Norway. J Immigr Minor Health.

[6] Vaez M, Rylander G, Nygren A, Asberg M, Alexanderson K (2007). Sickness absence and disability pension in a cohort of employees initially on long-term sick leave due to psychiatric disorders in Sweden. Soc Psychiatry Psychiatr Epidemiol.

[7] Beckman A, Hakansson A, Rastam L, Lithman T, Merlo J (2006). The role country of birth plays in receiving disability pensions in relation to patterns of health care utilisation and socioeconomic differences: a multilevel analysis of Malmo, Sweden. BMC public health.

[8] Werlen L, Helgesson M, Mittendorfer-Rutz E (2017). Differences in predictors of permanent work disability between immigrants and natives: a cohort study of adults with sick leave due to common mental disorders. BMJ Open.

[9] Ng E, Sanmartin C, Tu JV, Manuel DG (2015). All-cause and circulatory disease-related hospitalization, by generation status: evidence from linked data. Health Rep.

[10] Liddell BJ, Nickerson A, Sartor L, Ivancic L, Bryant RA (2016). The generational gap: mental disorder prevalence and disability amongst first and second generation immigrants in Australia. J Psychiatr Res.

[11] Allebeck P, Mastekaasa A (2004). Swedish council on technology assessment in health care (SBU). Chapter 5. Risk factors for sick leave - general studies. Scand J Public Health Suppl..

[12] Warner DF, Brown TH (2011). Understanding how race/ethnicity and gender define age-trajectories of disability: an intersectionality approach. Soc Sci Med.

[13] Vahtera J, Laine S, Virtanen M, Oksanen T, Koskinen A, Pentti J (2010). Employee control over working times and risk of cause-specific disability pension: the Finnish public sector study. Occup Environ Med.

[14] Ishtiak-Ahmed K, Perski A, Mittendorfer-Rutz E (2014). Risk markers of all-cause and diagnosis-specific disability pension - a prospectve cohort study of individuals sickness absent due to stress-related mental disorders. BMC Public Health.

[15] Mantyniemi A, Oksanen T, Salo P, Virtanen M, Sjosten N, Pentti J (2012). Job strain and the risk of disability pension due to musculoskeletal disorders, depression or coronary heart disease: a prospective cohort study of 69,842 employees. Occup Environ Med.

[16] Organisation for Economic Co-operation and Development (OECD). Sickness, Disability and Work: Breaking the Barriers. A synthesis of findings across OECD countries. Paris: OECD Publishing; 2010.

[17] Cantor-Graae E, Selten JP (2005). Schizophrenia and migration: a meta-analysis and review. Am J Psychiatry.

[18] Di Thiene D, Alexanderson K, Tinghog P, La Torre G, Mittendorfer-Rutz E (2015). Suicide among first-generation and second-generation immigrants in Sweden: association with labour market marginalisation and morbidity. J Epidemiol Community Health.

[19] van Oeffelen AA, Vaartjes I, Stronks K, Bots ML, Agyemang C (2013). Incidence of acute myocardial infarction in first and second generation minority groups: does the second generation converge towards the majority population?. Int J Cardiol.

[20] Gilliver SC, Sundquist J, Li X, Sundquist K (2014). Recent research on the mental health of immigrants to Sweden: a literature review. Eur J Pub Health.

[21] Behrenz L, Hammarstedt M, Månsson J (2007). Second-generation immigrants in the Swedish labour market. Int Rev Appl Econ.

[22] Crul M, Schneider J, Lelie F. The European second generation compared. Does the integration context matter? Amsterdam: Amsterdam University Press; 2012.

[23] Claussen B, Dalgard OS, Bruusgaard D (2009). Disability pensioning: can ethnic divides be explained by occupation, income, mental distress, or health?. Scand J Public Health..

[24] Sweden S (2010). Multi-generation register.

[25] The Swedish Social Insurance Agency. Social Insurance in figures 2016. Sweden; 2016.

[26] Hjern A (2012). Migration and public health: health in Sweden: the National Public Health Report 2012. Chapter 13. Scand J Public Health.

[27] Hjern A, Allebeck P (2002). Suicide in first- and second-generation immigrants in Sweden: a comparative study. Soc Psychiatry Psychiatr Epidemiol.

[28] Termorshuizen F, Wierdsma AI, Visser E, Drukker M, Sytema S, Laan W (2012). Psychosis and suicide risk by ethnic origin and history of migration in the Netherlands. Schizophr Res.

[29] World Health Organization (WHO). International Statistical Classification of Diseases and Related Health Problems, 10th revision (ICD 10). Geneva: WHO; 2010.

[30] Alexanderson K, Norlund A (2004). Swedish council on technology assessment in health care (SBU). Chapter 1. Aim, background, key concepts, regulations, and current statistics. Scand J Public Health Suppl.

[31] Osterberg T, Gustafsson B (2006). Disability pension among immigrants in Sweden. Soc Sci Med.

[32] Kluge U, Bogic M, Devillé W, Greacen T, Dauvrin M, Dias S (2012). Health services and the treatment of immigrants: data on service use, interpreting services and immigrant staff members in services across Europe. European Psychiatry.

[33] Li X, Sundquist J, Zoller B, Bennet L, Sundquist K (2013). Risk of hospitalization for type 2 diabetes in first- and second-generation immigrants in Sweden: a nationwide follow-up study. J Diabetes Complicat.

[34] Mousavi SM, Forsti A, Sundquist J, Hemminki K (2013). Ethnic differences in breast cancer risk and survival: a study on immigrants in Sweden. Acta Oncol.

[35] Ahonen EQ, Benavides FG, Benach J (2007). Immigrant populations, work and health-a systematic literature review. Scand J Work Environ Health.

[36] Helgesson M, Johansson B, Nordqvist T, Lundberg I, Vingard E (2013). Unemployment at a young age and later sickness absence, disability pension and death in native Swedes and immigrants. Eur J Pub Health.

[37] Alden L, Hammarstedt M. Integration of immigrants on the Swedish labour market - recent trends and explanations. Centre for Labour Market and Discrimination Study, Linnaeus University, Växjö, Sweden. 2014.

[38] Markaki Y. Do labour market conditions shape immigrant native gaps in employment outcomes? A comparison of 19 European countries. Colchester: Institute for Social and Economic Research University of Essex; 2014.

[39] Simon GE, Barber C, Birnbaum HG, Frank RG, Greenberg PE, Rose RM (2001). Depression and work productivity: the comparative costs of treatment versus nontreatment. J Occup Environ Med.

[40] Bhugra D, Gupta S, Schouler-Ocak M, Graeff-Calliess I, Deakin NA, Qureshi A (2014). EPA guidance mental health care of migrants. Eur Psychiatry.

[41] Sundquist K, Li X (2006). Coronary heart disease risks in first- and second-generation immigrants in Sweden: a follow-up study. J Intern Med.

[42] Organisation for Economic Co-operation and Development (OECD). Equal Opportunities? The labour market integration of the children if immigrants. Paris: OECD Publishing; 2010.

[43] Swedish Social Insurance Agency. Social insurance in figures 2014. Sweden; 2014.

[44] Saraiva Leao T, Sundquist J, Johansson LM, Johansson SE, Sundquist K (2005). Incidence of mental disorders in second-generation immigrants in sweden: a four-year cohort study. Ethnicity & health.

[45] Marmot M, Adelstein A, Bulusu L (1984). Lessons from the study of immigrant mortality. Lancet.

[46] Ludvigsson JF, Andersson E, Ekbom A, Feychting M, Kim JL, Reuterwall C (2011). External review and validation of the Swedish national inpatient register. BMC Public Health.

[47] Rahman S, Alexanderson K, Jokinen J, Mittendorfer-Rutz E (2014). Risk factors for suicidal behaviour in individuals on disability pension due to common mental disorders - a nationwide register-based prospective cohort study in Sweden. PLoS One.

[48] Ljungdahl LO, Bjurulf P (1991). The accordance of diagnoses in a computerized sick-leave register with doctor's certificates and medical records. Scand J Soc Med.

[49] Bockerman P, Hyytinen A, Maczulskij T (2016). Devil in disguise: does drinking lead to a disability pension?. Prev Med.

